# Hypertension Risk in Young Women With Polycystic Ovary Syndrome: A Nationwide Population-Based Cohort Study

**DOI:** 10.3389/fmed.2020.574651

**Published:** 2020-09-23

**Authors:** Cheng-Hsuan Wu, Lu-Ting Chiu, Yu-Jun Chang, Chun-I Lee, Maw-Sheng Lee, Tsung-Hsien Lee, James Cheng-Chung Wei

**Affiliations:** ^1^Institute of Medicine, Chung Shan Medical University, Taichung, Taiwan; ^2^Women's Health Research Laboratory, Changhua Christian Hospital, Changhua, Taiwan; ^3^School of Medicine, Kaohsiung Medical University, Kaohsiung, Taiwan; ^4^Management Office for Health Data, Clinical Trial Research Center, China Medical University Hospital, Taichung, Taiwan; ^5^Epidemiology and Biostatistics Center, Changhua Christian Hospital, Changhua, Taiwan; ^6^Department of Obstetrics and Gynecology, Chung Shan Medical University Hospital, Taichung, Taiwan; ^7^Division of Infertility, Lee Women's Hospital, Taichung, Taiwan; ^8^Department of Allergy, Immunology and Rheumatology, Chung Shan Medical University Hospital, Taichung, Taiwan; ^9^Graduate Institute of Integrated Medicine, China Medical University, Taichung, Taiwan

**Keywords:** hypertension, polycystic ovarian syndrome, population-based cohort study, young women, comorbidities

## Abstract

**Objective:** A number of publications have assessed the prevalence of hypertension in polycystic ovary syndrome (PCOS) patients with inconclusive results. Since in general populations the occurrence of hypertension is related to age and comorbidities, we investigated the incidence rate and hazard ratios (HRs) of hypertension between healthy subjects and young women with PCOS as well as comorbidities.

**Methods:** We conducted a population-based retrospective cohort study by using the National Health Insurance Research Database in Taiwan. The cohort included women with the diagnosis of PCOS between 2000 and 2012. Those without PCOS were selected as the control group at a ratio of 4:1 by an age-matched systematic random-sampling method. Cox proportional hazard regression analysis was used to determine the effects of PCOS on the risks of developing hypertension. Stratification analysis was performed to elucidate the interaction among PCOS and the comorbidities, which affect the incidence of hypertension.

**Results:** The PCOS cohort consisted of 20,652 patients with PCOS and the comparison cohort consisted of 82,608 matched patients without PCOS. There was no difference in the distribution of age between the PCOS cohort and the comparison cohort (29.1 ± 6.8 vs. 29.0 ± 6.5, *p* = 0.32). The incidence rates of hypertension were 7.85 and 4.23 per 1,000 person-years in the PCOS and comparison groups, respectively. A statistically significant higher risk of hypertension was found in the PCOS cohort (adjusted HR = 1.62, 95% confidence interval = 1.48–1.76) than in the comparison cohort. After a joint analysis of comorbidities, the adjusted HR of hypertension was 9.44 (95% confidence interval = 7.27–12.24) for PCOS patients with comorbidities of diabetes mellitus (DM) and hyperlipidemia compared with women with neither PCOS nor DM and hyperlipidemia.

**Conclusion:** The risk of developing hypertension in young women with PCOS was higher than in controls in this cohort study. The comorbidities of DM and hyperlipidemia could interact with PCOS patients and further increase the risk of hypertension. An earlier screening for hypertension and comorbidities in patients with PCOS may be warranted, even in young women.

## Introduction

Polycystic ovary syndrome (PCOS) is characterized by ovulatory dysfunction, hyperandrogenism, and polycystic ovarian morphology when other causes are excluded according to the Rotterdam criteria (1). PCOS is the most common endocrine disease affecting the women of reproductive age. Insulin resistance (IR) and androgen excess are prevalent in patients with PCOS (2, 3). Previous studies have demonstrated that almost all cardiovascular risk factors including IR, diabetes mellitus (DM), dyslipidemia, obesity, hypertension, and metabolic syndrome are associated with PCOS (4–8). These risk factors are present even in young patients with PCOS and predispose to the development of endothelial dysfunction, early atherosclerosis and cardiovascular disease (CVD) (9–12).

Patients with PCOS, especially with hyperandrogenic phenotype, are exposed to several cardiometabolic risk factors that increase their chance for developing hypertension (13). Androgen excess in PCOS may also directly affect the vascular properties of arterial walls involved in the atherogenic process (14). Although hypertension represents one of the main cardiovascular risk factors in general populations, previous studies have shown inconsistent results on hypertension in PCOS. Recently, a meta-analysis confirmed a greater risk of developing hypertension in patients with PCOS but demonstrated that this risk is increased only in the women of reproductive age with PCOS (15). However, another systemic review and meta-analysis showed that no significant difference for hypertension between non-obese women with PCOS and controls (16).

DM, dyslipidemia, asthma, chronic kidney disease (CKD), and CVD are also some comorbidities that are prevalent in the patients with hypertension (17–21). In addition, given the multitude of comorbidities associated with PCOS, such as DM (16), dyslipidemia (22), CKD (23), asthma (24), chronic obstructive pulmonary disease (COPD) (25), and CVD (9, 11), the objective of this study is to investigate the risk of hypertension associated with the women of reproductive age with PCOS and the abovementioned comorbidities in a population-based cohort database.

## Materials and Methods

### Data Source

This is a retrospective population-based cohort study. The Taiwan government launched the National Health Insurance program in 1995, which contains more than 99% Taiwanese residents through a universal single-payer health system. In this study, we used the data of the Longitudinal Health Insurance Database 2000 (LHID 2000), which consists of registration files and original claims data from 1996 to 2013 of 1 million patients randomly selected from the National Health Insurance Research Database (NHIRD). Meanwhile, many studies have provided validation to the database (9, 26, 27). As all personal information is encrypted with de-identification process for research purposes in NHIRD database, no patient's consent was required. The LHID identifies diseases were based on the International Classification of Diseases, Ninth Revision, Clinical Modification (ICD-9-CM). This study was approved by the Research Ethics Committee of China Medical University and Hospital in Taiwan (CMUH-104-REC2-115-CR-4).

### Study Population

**Figure 1:**
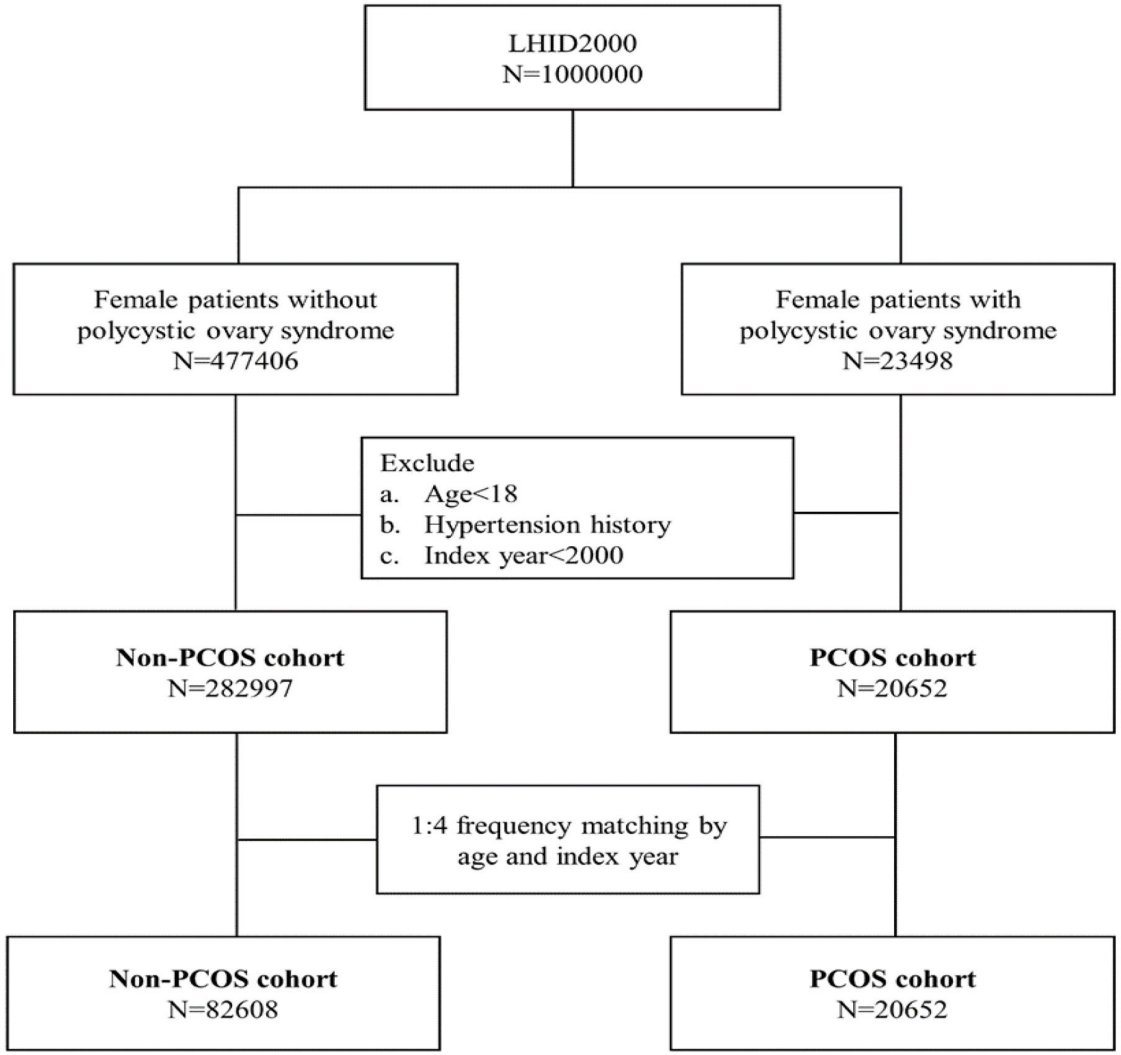
Flow chart of the subject selection process.

Patients who were diagnosed with PCOS (ICD-9-CM 256.4) were recruited in this cohort study between January 1, 2000, and December 31, 2012. We excluded patients who were diagnosed with PCOS based on A code: A189, not ICD-9-CM code: 256.4 between January 1, 1996, and December 31, 1999. We defined the diagnosis date of PCOS as the index date. The exclusion criteria included the followings: (1). age <18 years old, (2). hypertension history, (3). PCOS index year <2000. Female patients without PCOS during the study period were randomly selected from the same database for the comparison cohort. The comparison cohort was frequency-matched with the PCOS cohort at a 4:1 ratio according to age (every 5 years) and the index year. The index date for the comparison cohort was randomly assigned. Figure 1 illustrates the selection procedure.

The event of interest in the study was a new diagnosis of hypertension (ICD-9-CM codes 401–405) with at least two outpatient visits or at least one hospitalization record from January 1, 2000 to December 31, 2013. Each patient was monitored from the index year until their diagnosis with hypertension; withdrawal from insurance; or at the end of December 31, 2013, whichever came first.

Comorbidities that could be potentially associated with PCOS and hypertension were included as follows: diabetes mellitus (DM; ICD-9-CM code 250), hyperlipidemia (ICD-9-CM code 272), chronic obstructive pulmonary disease (COPD; ICD-9-CM code 490-492, 494 and 496), asthma (ICD-9-CM code 493), chronic kidney disease (CKD; ICD-9-CM 250.4, 274.1, 283.11, 403, 404, 440.1, 442.1, 447.3, 572.4, 580–588, 642.1, and 646.2), coronary artery disease (CAD; ICD-9-CM code 410-414), and congestive heart failure (CHF; ICD-9-CM code 398.91, 402.01, 402.11, 402.91, 404.01, 404.03, 404.11, 404.13, 404.91, 404.91, 404.93, and 428).

### Statistical Analysis

We described the age and follow-up time by mean with standard deviation and tested the difference between the PCOS and comparison cohorts by using the *t*-test. The categorical variables were presented as the numbers with percentage and tested the difference by using the Chi-square test. The hazard ratio (HR) with 95% confidence interval (CI) for hypertension was estimated by Cox proportional regression analysis, which was adjusted for the potential confounding variables, such as age and comorbidities between the PCOS cohort and comparison cohorts. Stratification analysis was performed to elucidate the interaction among PCOS and the comorbidities, which affect the incidence of hypertension. To further clarify the correlation between PCOS with comorbidities and hypertension, we subsequently conducted a joint analysis of DM, hyperlipidemia, and PCOS, and compared the risk of hypertension in each combination. In addition, we measured and compared the cumulative incidence curves for hypertension between the PCOS cohort and tested the difference of the curves by using the log rank test. All statistical analyses were performed by using SAS version 9.4 (Version 9.4, SAS Institute Inc., Cary, NC, USA). The significance level for statistical analysis was set at *P* < 0.05.

## Results

The PCOS cohort consisted of 20,652 patients with PCOS and the comparison cohort consisted of 82,608 matched patients without PCOS (Table 1). There was no difference in the distribution of age between the PCOS cohort and the comparison cohort (29.0 ± 6.5 vs. 29.1 ± 6.8, *p* = 0.32). Patients with PCOS tended to have a higher proportion of DM, hyperlipidemia, COPD, asthma, CKD, and CAD than those in the control cohort (*p* < 0.0001). The mean follow-up periods were similar in the two cohorts (4.9 ± 3.5 years vs. 4.8 ± 3.5 years, *p* = 0.19).

**Table 1:** Baseline characteristics of patients with and without polycystic ovarian syndrome.

	**Polycystic ovarian syndrome**		***p*-value**
	**No (*n* = 82,608)**	**Yes (*n* = 20,652)**	
**Age (years)**, ***n*** **(%)**			1.00
≤30	48,656 (58.9)	12,164 (58.9)	
>30	33,952 (41.1)	8,488 (41.1)	
Mean (SD)	29.1 (6.8)	29.0 (6.5)	0.32
**Follow-up time (years)**			
Mean (SD)	4.9 (3.5)	4.8 (3.5)	0.19
**Comorbidity**, ***n*** **(%)**			
Diabetes mellitus	1,218 (1.5)	1,048 (5.1)	<0.0001
Hyperlipidemia	1,858 (2.3)	1,126 (5.5)	<0.0001
COPD	10,092 (12.2)	3,302 (16.0)	<0.0001
Asthma	4,440 (5.4)	1,480 (7.2)	<0.0001
Chronic kidney disease	975 (1.2)	363 (1.8)	<0.0001
Coronary artery disease	781 (1.0)	118 (0.6)	<0.0001
Congestive heart failure	120 (0.2)	20 (0.1)	0.19

*COPD, chronic obstructive pulmonary disease; SD, standard deviation*.

**Figure 2:**
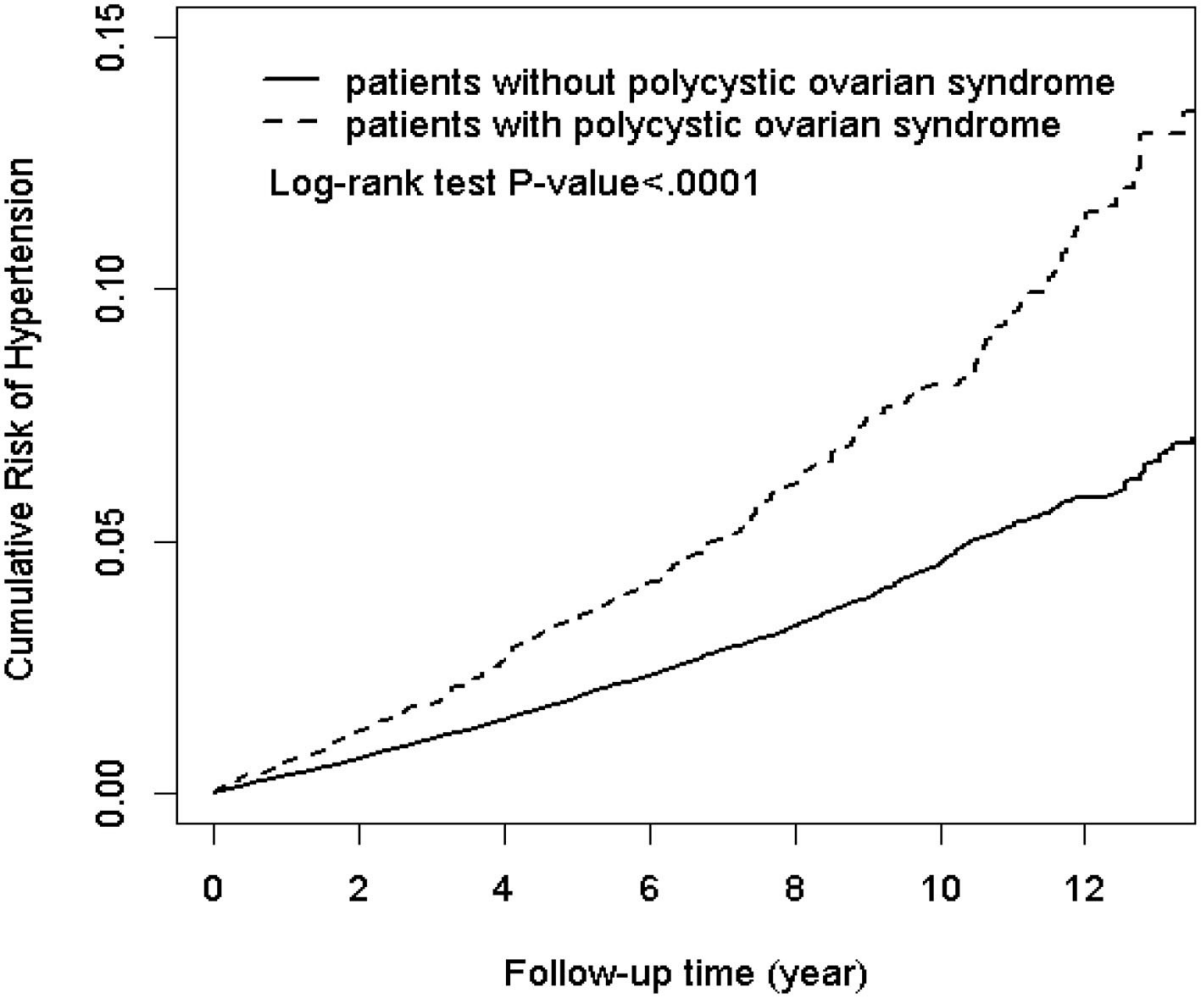
Kaplan-Meier plot of the cumulative risk of hypertension in patients with and without polycystic ovarian syndrome (log-rank < 0.0001).

The results of the Kaplan-Meier analysis showed that the PCOS cohort had a significantly higher cumulative incidence of hypertension than the comparison cohort (log-rank test, *p* < 0.001) at the end of follow-up (Figure 2). The overall incidence rates of hypertension in the PCOS cohort and the comparison cohort were 7.85 and 4.23 per 1,000 person-years, respectively. After adjusting for age and all comorbidities, the risk of hypertension was significantly higher in the patients with PCOS than in the comparison cohort by an adjusted HR of 1.62 (95% CI = 1.48–1.76, *p* < 0.001). The incidence of hypertension increases with age. Compared with patients aged ≤30 years, the risk of hypertension occurrence was 2.88-fold higher in patients aged above 30 years (95% CI = 2.65–3.13, *p* < 0.001). As for potential comorbidities, patients with DM (adjusted HR = 3.00; 95% CI = 2.61–3.45), hyperlipidemia (adjusted HR = 2.42; 95% CI = 2.11–2.79), asthma (adjusted HR = 1.27; 95% CI = 1.04–1.56), CKD (adjusted HR = 1.53; 95% CI = 1.20–1.95), and CAD (adjusted HR = 1.82; 95% CI = 1.42–2.33) are at a relatively higher risk of developing hypertension than patients without comorbidities (Table 2).

**Table 2:** The incidence and HRs for risk of hypertension associated with PCOS, age and comorbidities.

**Variables**	**Hypertension (*****n*** **=** **2,505)**			**Crude HR (95% CI)**	**Adjusted HR (95% CI)**
	**Event**	**PY**	**IR**		
**PCOS**					
No	1,715	405,264	4.23	1 (reference)	1 (reference)
Yes	790	100,597	7.85	1.85 (1.70–2.02)^**^	1.62 (1.48–1.76)^**^
**Age, years**					
≤30	881	316,850	2.78	1 (reference)	1 (reference)
>30	1624	189,011	8.59	3.16 (2.91–3.44)^**^	2.88 (2.65–3.13)^**^
**Comorbidities**					
Diabetes mellitus					
No	2,229	495,115	4.50	1 (reference)	1 (reference)
Yes	276	10,746	25.68	5.79 (5.11–6.56)^**^	3.00 (2.61–3.45)^**^
Hyperlipidemia					
No	2,229	493,256	4.52	1 (reference)	1 (reference)
Yes	276	12,604	21.90	5.06 (4.46–5.73)^**^	2.42 (2.11–2.79)^**^
COPD					
No	2125	450,277	4.72	1 (reference)	1 (reference)
Yes	380	55,584	6.84	1.51 (1.35–1.68)^**^	1.10 (0.96–1.27)
Asthma					
No	2338	483,376	4.84	1 (reference)	1 (reference)
Yes	167	22,485	7.43	1.62 (1.38–1.89)^**^	1.27 (1.04–1.56)^*^
CKD					
No	2,437	500,077	4.87	1 (reference)	1 (reference)
Yes	68	5,784	11.76	2.47 (0.94–3.15)^**^	1.53 (1.20–1.95)^**^
CAD					
No	2,439	501,290	4.87	1 (reference)	1 (reference)
Yes	66	4,571	14.44	2.96 (2.32–3.78)^**^	1.82 (1.42–2.33)^**^
CHF					
No	2,496	505,274	4.94	1 (reference)	1 (reference)
Yes	9	587	15.33	3.21 (1.67–6.18)^**^	1.65 (0.85–3.20)

* *p < 0.05*,

** *p < 0.001*.

*HR adjusted for age, diabetes mellitus, hyperlipidemia, COPD, asthma, CKD, CAD and CHF*.

*CAD, coronary artery disease; CHF, congestive heart failure; CI, confidence interval; CKD, chronic kidney disease; COPD, chronic obstructive pulmonary disease; HR, hazard ratio; IR, incidence rate, per 1,000 person-years; PCOS, polycystic ovarian syndrome; PY, person-years*.

The HRs of hypertension in PCOS and control group by stratification analysis with various comorbidities were demonstrated in Table 3. It is interesting to note that the risk of hypertension in patients with PCOS is more prominent at young age (≤30 years, adjusted HR: 2.20, 95% CI = 1.91–2.54, *p* < 0.001; whereas for patients older than 30 years, adjusted HR: 1.30, 95% CI = 1.17–1.46, *p* < 0.001). When stratified by comorbidity, several significantly increased risks of hypertension were found in the following group of patients with PCOS: patients with and without DM, hyperlipidemia, COPD, and asthma. Nevertheless, CKD, CAD, and CHF did not change the HR for the incidence of hypertension in the patients with PCOS (Table 3).

**Table 3:** The incidence and HRs for risk of hypertension in patients with and without PCOS stratified by age and comorbidities.

**Variables**	**PCOS**						**Crude HR (95% CI)**	**Adjusted HR (95% CI)**
	**No**			**Yes**				
	**Event**	**PY**	**IR**	**Event**	**PY**	**IR**		
**Total**	1,715	405,264	4.23	790	100,597	7.85	1.85 (1.70–2.02)^**^	1.62 (1.48–1.76)^**^
**Age, years**								
≤30	528	253,795	2.08	353	63,055	5.60	2.69 (2.35–3.08)^**^	2.20 (1.91–2.54)^**^
>30	1,187	151,469	7.84	437	37,542	11.64	1.48 (1.33–1.66)^**^	1.30 (1.17–1.46)^**^
**Comorbidities**								
Diabetes mellitus								
No	1,599	399,417	4.00	630	95,698	6.58	1.64 (1.50–1.80)^**^	1.55 (1.42–1.70)^**^
Yes	116	5847	19.84	160	4,899	32.66	1.65 (1.30–2.10)^**^	1.71 (1.34–2.19)^**^
Hyperlipidemia								
No	1,574	397,525	3.96	655	95,732	6.84	1.72 (1.57–1.89)^**^	1.61 (1.47–1.77)^**^
Yes	141	7,739	18.22	135	4,865	27.75	1.52 (1.20–1.92)^**^	1.49 (1.18–1.89)^*^
COPD								
No	1,472	363,493	4.05	653	86,784	7.52	1.86 (1.69–2.04)^**^	1.63 (1.49–1.79)^**^
Yes	243	41,771	5.82	137	13,813	9.92	1.69 (1.37–2.09)^**^	1.49 (1.20–1.85)^**^
Asthma								
No	1,615	388,361	4.16	723	95,015	7.61	1.83 (1.67–2.00)^**^	1.61 (1.47–1.76)^**^
Yes	100	16,903	5.92	67	5,582	12.00	2.01 (1.47–2.74)^**^	1.58 (1.14–2.18)^*^
CKD								
No	1,661	401,064	4.14	776	99,013	7.84	1.89 (1.74–2.06)^**^	1.66 (1.52–1.81)^**^
Yes	54	4,200	12.86	14	1,584	8.84	0.65 (0.36–1.18)	0.64 (0.34–1.11)
CAD								
No	1,664	401,369	4.15	775	99,920	7.76	1.88 (1.72–2.04)^**^	1.63 (1.49–1.78)^**^
Yes	51	3894	13.10	15	676	22.19	1.70 (0.96–3.03)	1.60 (0.89–2.85)
CHF								
No	1,709	404,767	4.22	787	100,506	7.83	1.85 (1.71–2.02)^**^	1.64 (1.50–1.79)^**^
Yes	6	497	12.07	3	90	33.33	2.70 (0.65–11.32)	3.55 (0.69–18.12)

* *p < 0.01*,

** *p < 0.001*.

*HR adjusted for age, diabetes mellitus, hyperlipidemia, COPD, asthma, CKD, CAD and CHF*.

*CAD, coronary artery disease; CHF, congestive heart failure; CI, confidence interval; CKD, chronic kidney disease; COPD, chronic obstructive pulmonary disease; HR, hazard ratio; IR, incidence rate, per 1,000 person-years; PCOS, polycystic ovarian syndrome; PY, person-years*.

Among the comorbidities, DM alone was associated with hypertension development with an adjusted HR of 3.19 (95% CI = 2.52–4.04, *p* < 0.001) (Table 4). The adjusted HR of hypertension increased to 6.62 for PCOS patients with DM compared with women with neither PCOS nor comorbidities of DM and hyperlipidemia. The corresponding adjusted HR of hypertension even increased to 9.44 (95% CI = 7.27–12.24, *p* < 0.001) for PCOS patients with DM and hyperlipidemia (Table 4).

**Table 4:** The incidence and HRs for risk of hypertension associated with PCOS, diabetes and hyperlipidemia.

**PCOS**	**DM**	**HPL**	**N**	**Event**	**PY**	**IR**	**Crude HR (95% CI)**	**Adjusted HR (95% CI)**
N	N	N	79,882	1,501	393,156	3.82	1 (reference)	1 (reference)
Y	N	N	18,812	555	92,222	6.02	1.57 (1.43–1.73)^*^	1.58 (1.44–1.74)^*^
N	Y	N	868	73	4,368	16.71	4.39 (3.47–5.55)^*^	3.19 (2.52–4.04)^*^
N	N	Y	1,508	98	6,261	15.65	4.29 (3.49–5.26)^*^	3.11 (2.53–3.82)^*^
Y	Y	N	714	100	3,509	28.50	7.54 (6.16–9.23)^*^	6.62 (5.40–8.12)^*^
Y	N	Y	792	75	3,475	21.58	5.87 (4.65–7.40)^*^	4.39 (3.48–5.54)^*^
N	Y	Y	350	43	1,478	29.09	7.95 (5.87–10.77)^*^	5.12 (3.77–6.95)^*^
Y	Y	Y	334	30	1,389	21.60	11.93 (9.22–15.45)^*^	9.44 (7.27–12.24)^*^

* *p < 0.001*.

*HR adjusted for age, chronic obstructive pulmonary disease, asthma, chronic kidney disease, coronary artery disease, and congestive heart failure*.

*CI, confidence interval; DM, diabetes mellitus; HPL, hyperlipidemia; HR, hazard ratio; IR, incidence rate, per 1,000 person-years; PCOS, polycystic ovarian syndrome; PY, person-years*.

## Discussion

We conducted this population-based cohort study based on a large size of young female population with PCOS at an average age of 29 years. Young women are generally at a low risk of hypertension. Nevertheless, this study confirms that the risk of developing hypertension in young female population with PCOS is 62% higher than those without PCOS (adjusted HR = 1.62, 95% CI = 1.48–1.76). Meanwhile, the comorbidities of DM and hyperlipidemia were also related with the risk of hypertension and could interact with women having PCOS, further increasing the risk of hypertension.

Previous studies on the relations between PCOS and hypertension remain debatable. Recently, a cross-sectional study in Brazil found the prevalence of hypertension at 65% among young patients with PCOS (mean age: 25–26.5 years) vs. 41% among control women without PCOS (mean age: 29 years) (28). A meta-analysis study revealed a greater risk of hypertension in women with PCOS but demonstrated that this pooled relative risk is only increased in the women of reproductive age with PCOS (1.70-fold, 95% CI: 1.43–2.07) (15). The abovementioned results are consistent with our findings. We found an adjusted HR of 1.62 for developing hypertension after controlling for age and other comorbidities. However, another systemic review observed no significant difference for hypertension between non-obese women of reproductive age with and without PCOS (16). A possible explanation for this discrepancy is that body mass index (BMI) was not included for analysis in our study. Obesity is considered as a key factor for the alteration of blood pressure in women with PCOS (29, 30).

The mechanism underlying the increased prevalence of hypertension in PCOS has been linked to several factors such as IR and hyperinsulinemia (31), hyperandrogenism (8), obesity (30), and heart autonomic dysfunction (32). IR interferes with the endothelium-dependent vasodilatation mechanisms causing vascular muscle wall hypertrophy and compensatory hyperinsulinemia further contributes to the development of hyperandrogenemia (33). The hyperandrogenic state of PCOS develops exacerbated cardio-metabolic profile with consequent endothelial dysfunction and elevated blood pressure (34). Additionally, IR-related compensatory hyperinsulinemia may affect blood pressure through an imbalance of autonomic nervous system, decreased production of nitric oxide, and an increased reabsorption of renal sodium (28).

Our results found that patients with PCOS and patients with hypertension share similar characteristics of chronic comorbidities (35, 36). Patients with PCOS tended to have a higher proportion of DM, hyperlipidemia, COPD, asthma, CKD, and CAD than those in the control cohort (*p* < 0.0001) (Table 1). Meanwhile, patients with DM, hyperlipidemia, asthma, or CKD had a relatively higher risk of developing hypertension than patients without comorbidities (Table 2). DM or hyperlipidemia alone was a stronger risk factor than PCOS alone in association with the development of hypertension (adjusted HR: 3.00, 2.42, and 1.62, respectively) (Table 2). After stratification analysis, women with hyperlipidemia, COPD, asthma, CKD, or CAD did not further modify the hypertension risk in PCOS patients than in controls. By contrast, women with DM could further increase the risk of hypertension in patients with PCOS (Table 3).

Age is another substantial risk factor for the manifestation of hypertension. In this population-based analysis, the risk of hypertension occurrence was 2.88-fold (95% CI = 2.65–3.13, *p* < 0.001) higher in patients aged above 30 years as compared with the patients aged ≤30 years (Table 2). After stratification analysis, it is interesting to find that an early diagnosis of PCOS at a younger age (≤30 years) increased the incidence of hypertension (Table 3). This finding is the same as the one demonstrated by the log-rank test. Therefore, earlier screening for hypertension in patients with PCOS may be warranted, even in young women.

This study highlighted the risk of developing hypertension in the women of reproductive age with PCOS comorbid with DM and hyperlipidemia by an adjusted HR of 9.44 (95% CI = 7.27–12.24, *p* < 0.001) (Table 4). Therefore, once the diagnosis of PCOS is made, there should be an assessment of metabolic status and comorbidities (37). Recently, a systemic study summarized the findings of 16 studies and found a greater risk of CVD in the women of reproductive age with PCOS (5). According to the recently published international PCOS guidelines, the global CVD risk should be routinely assessed in women with PCOS (38). There should be an annual monitoring of glucose, lipid profiles, blood pressure, and weight in these women. All providers engaged in the multidimensional care of women with PCOS should be alarmed of these long-term health risks to provide appropriate screening, counseling, and management options (12).

This retrospective study was performed based on the database NHIRD, which contained all the medical record information of one million Taiwanese randomly selected from the 23 million Taiwan population. All the incomplete data in the medical records for medical cost claim are audited by the authority National Health Insurance Administration. Several limitations to our study should be considered. First, the NHIRD does not provide baseline information on patients, such as BMI, smoking, alcohol consumption, and family history. These are all the risk factors for the development of hypertension. Thus, we were unable to control for these potentially confounding factors. Second, parity is also not recorded in the database. A recent study has reported that nulliparity is associated with a higher risk of pregnancy-associated hypertension (39). Third, we did not compare the different PCOS phenotypes, which may have potentially affected our results. Fourth, this study includes the retrospective design and prospective cohort studies based on the homogeneous populations of large sample size are still needed to verify our results.

## Conclusion

In conclusion, our nationwide population-based cohort study provided evidence of an increased risk of hypertension in the women of reproductive age with PCOS. The early diagnosis of PCOS of younger age corresponds to the higher cumulative incidence of hypertension. The comorbidities with DM and hyperlipidemia further increase the risk of developing hypertension. An earlier screening for hypertension and comorbidities in patients with PCOS may be warranted, even in young women.

## Data Availability Statement

All datasets generated for this study are included in the article/supplementary material.

## Ethics Statement

The studies involving human participants were reviewed and approved by Research Ethics Committee of China Medical University and Hospital in Taiwan (CMUH-104-REC2-115-CR-4). Written informed consent for participation was not required for this study in accordance with the national legislation and the institutional requirements.

## Author Contributions

C-HW and T-HL: conceptualization. T-HL, C-IL, and M-SL: methodology. T-HL and M-SL: validation. L-TC and Y-JC: formal analysis. C-IL: resources. C-HW: writing—original draft preparation. T-HL and JW: writing—review and editing. JW: funding acquisition. All authors have read and agreed to the published version of the manuscript.

## Conflict of Interest

The authors declare that the research was conducted in the absence of any commercial or financial relationships that could be construed as a potential conflict of interest.
